# Seroprevalence of SARS-CoV-2 in wet market workers in Dhaka, Bangladesh in 2022

**DOI:** 10.1016/j.ijregi.2024.100497

**Published:** 2024-11-24

**Authors:** Mahbubur Rahman, Ahmed Nawsher Alam, Sudipta Sarkar, Manjur Hossain Khan, Punam Mangtani, Saira Butt, Anne Conan, Damer Blake, Fiona Tomley, Guillaume Fournie, Tahmina Shirin, Patrick Nguipdop-Djomo

**Affiliations:** 1 Institute of Epidemiology, Disease Control and Research (IEDCR), Dhaka, Bangladesh; 2 London School of Hygiene and Tropical Medicine (LSHTM), London, UK; 3 Royal Veterinary College (RVC), London, UK; 4 City University of Hong Kong, Hong Kong, Hong Kong SAR; 5 French Agricultural Research Centre for International Development (CIRAD), UMR ASTRE, Harare, Zimbabwe; 6 ASTRE, Univ Montpellier, CIRAD, INRAE, Montpellier, France; 7 Université de Lyon, INRAE, VetAgro Sup, UMR EPIA, Clermont-Auvergne-Rhône-Alpes, France; 8 Université Clermont Auvergne, INRAE, VetAgro Sup, UMR EPIA, Clermont-Auvergne-Rhône-Alpes, France

**Keywords:** SARS-Cov-2, Serosurvey, Bangladesh, Wet market, Occupational

## Abstract

**Objectives:**

We assessed the transmission of SARS-CoV-2 and vaccine receipt in a representative sample of wet market workers in a highly dense, low-income setting. Wet markets are key in many Asian settings, including Dhaka, Bangladesh, for fresh food, including animal protein.

**Methods:**

During early 2022, we assessed the prevalence of anti-SARS-CoV-2 antibodies in a random sample of poultry and vegetable workers in 15 wet markets, and investigated associations with socio-demographic characteristics and COVID-19 vaccination.

**Results:**

In the 204 poultry workers and 87 vegetable sellers recruited, COVID-19 vaccine uptake was similar (69% vs 67%). The most common vaccines were whole inactivated vaccines (Sinopharm or Sinovac; 44%). Nearly all (99%) participants were positive for anti-SARS-CoV-2 spike antibodies. Anti-SARS-CoV-2 nucleocapsid antibodies, indicative of recent infection, was 59.4% (95% confidence interval 54.7-64.0%). There was no evidence that the latter was associated any characteristics, except vaccination (adjusted odds ratio 0.49; 95% confidence interval 0.23-1.02; *P* = 0.093).

**Conclusions:**

The ubiquity of anti-spike and high prevalence of anti-nucleocapsid antibodies were consistent with high transmission after three previous pandemic waves. Wet markets have a potential role amplifying transmission of airborne pathogens, such as SARS-CoV-2 and Avian Influenza Viruses, and an important public-facing hotspot for close monitoring for future emerging infections.

## Highlights

• Wet markets are an important source of protein in low-income settings.
• Bangladesh wet market workers seem at a high risk of SARS-CoV-2 during the pandemic.
• Messenger RNA vaccines appear to be protective in this group based on anti-nucleocapsid antibodies.
• These frontline workers are a priority for emerging airborne pathogen surveillance.

## Introduction

Wet markets are important in many settings, including Bangladesh, as places where large segments of the population regularly source fresh animal protein [1,2] and other food items. These markets are often covered and usually crowded. The high population density (of animals and people) with frequent contact, limited waste management and biosecurity measures, and limited ventilation can facilitate transmission of infectious disease agents, including airborne pathogens such as SARS-CoV-2 [[3], [4], [5]].

At the height of the COVID-19 pandemic when restrictions to population movements and social distancing in Bangladesh were in place, wet markets remained open [5]. Non-pharmaceutical infection prevention and control measures such as social distancing and use of face coverings were also absent or rarely implemented in markets. Market workers appeared to be at higher risk of exposure to SARS-CoV-2 than the average population due to crowded working conditions and because they mostly belong to a population group with low a socioeconomic status [6,7].

During a study to assess the risk of avian influenza virus spillover from birds to humans in Dhaka's wet markets during February and March 2022, we measured the prevalence of anti-SARS-CoV-2 antibodies in market workers (poultry workers and vegetable sellers) and investigated its association to socio-demographic characteristics and COVID-19 vaccination history. To the best of our knowledge, this is the first study measuring prevalence of anti-SARS-CoV-2 antibodies in this occupational group.

## Methods

### Study setting and design

We conducted a cross-sectional study in wet markets in Dhaka, Bangladesh, with data collected in February and March 2022, during the pandemic's fourth wave in Bangladesh, when the Omicron SARS-CoV-2 variant was dominant [[8], [9], [10]]. Bangladesh had rolled out its vaccination drive for frontline health workers on January 27, 2021 and for the general population on February 7, 2021, administrating the UK Oxford–AstraZeneca vaccine (ChAdOx1-S [recombinant] vaccine). From June 19, 2021, Bangladesh started using a second vaccine, China's Sinopharm BIBP (whole inactivated virus vaccine). Subsequently, Bangladesh used Pfizer–BioNTech (messenger RNA [mRNA]–based COVID-19 vaccine) from June 21, 2021, Moderna (mRNA based COVID-19 vaccine) from July 13, 2021, and Sinovac (whole inactivated virus vaccine) from December 11, 2021 [11].

Participants were recruited in early 2022 using a two-stage self-weighted random sampling approach: first, 15 Live Poultry Markets were selected from those markets with >40 poultry stalls with a selection probability proportional to the number of poultry stalls in the market; next, market stalls were stratified as either poultry or vegetable/fruit stalls and were selected using simple random sampling. In each market, 13-14 poultry workers and 6-7 vegetable and fruit workers were invited to participate in the survey, with a maximum of two workers randomly selected per selected stalls.

### Data sources and data collection

Participants were asked to provide biological samples (a nasopharyngeal swab and 5 ml of peripheral venous blood) and respond to an interviewer-administered structured questionnaire consisting of closed questions. Direct observations were also made of hygiene conditions in the market and on the market stalls.

Each naso-pharyngeal swab was tested by multiplex reverse transcription–polymerase chain reaction (RT-PCR) for SARS-CoV-2 (Duplex E Gene) viruses [12]. Blood samples were processed to obtain sera, which were tested separately for anti-SARS-CoV-2 immunoglobulin (Ig)G antibodies against the spike (anti-S) and nucleocapsid (anti-N) proteins using the Abbott enzyme-linked immunosorbent assay (Abbott Diagnostics, Abbott Park, Illinois, USA) [13].

### Variables and data management

The outcomes for this analysis were for respiratory sample RT-PCR cycle threshold value ≤38.0 [12] and for serum samples positive results in SARS-CoV-2 anti-S and anti-N antibody assays according to manufacturer's instructions [13]. Anti-S IgG antibodies have a longer half-life than anti-N IgG antibodies and can be elicited by SARS-CoV-2 natural infection and all COVID-19 vaccinations [14]. They are longer lasting after natural infection and have been correlated with protection against disease [15]. Anti-N IgG antibodies are elicited by natural infection and have a much shorter half-life than anti-S antibodies [14,16]. They are not elicited by most COVID-19 vaccines (only whole virus—killed or attenuated, e.g. Sinopharm and Sinovac in the case of Bangladesh) and are regarded as a better proximate indicator of recent infection. The assays used did not allow a quantitative estimation of antibody titers.

Explanatory variables collected from the questionnaires included socio-demographic information (age, sex, religion, education, household crowding, role in the market stall) and other risk factors, such as tobacco smoking history, comorbidities (asthma and diabetes), facemask use, household COVID-19 cases, and COVID-19 vaccination history based on hand-held records.

### Statistical analysis

Questionnaire data were entered electronically using the Open Data Kit platform, then exported for cleaning, merging with laboratory results using participant unique barcodes, for analysis. After checks for consistency, age was transformed into a categorical variable (age groups); education, role in the market stall, tobacco smoking history, and vaccination history were regrouped into less categories to minimize data sparsity.

We tabulated the characteristics of study participants as a single group and as poultry workers vs vegetable sellers. We then computed the prevalence and 95% confidence intervals (CIs) of anti-S and anti-N antibodies as a single group, by worker group, and by individual characteristics, taking into account the cluster sampling design at the market level by using cluster-robust standard errors. We also assessed RT-PCR results for respiratory samples to explore any risk of active infection.

To account for the cluster sampling design at market level, we used univariable random-effects logistic regression models with market-level random intercepts to estimate the crude odds ratio (OR) and 95% CI for association between seropositivity and each participants’ socio-demographic characteristics and risk factors. We then fitted a multivariable random-effects model simultaneously, adjusting for all variables at the same time. Two multivariable models were run for overall vaccination status and type of vaccine separately to prevent multicollinearity between these two variables. The Wald test was used to obtain *P*-values.

All statistical analyses were done using Stata 18.

## Results

We recruited 291 participants (204 poultry workers and 87 vegetable/fruit workers) across 15 markets. All participants were men, and nearly all Muslim. Poultry workers were, on average, younger than vegetable sellers (78.5% aged under 40 years old, compared with 57.5%) and more educated (42.6% reporting secondary education or higher compared with 28.7%). Most vegetable/fruit workers owned their stall and were the sole worker, whereas 61.8% of poultry workers were employees. Smoking and household overcrowding were common in both worker groups. COVID-19 vaccine uptake was similar in both groups, with 68.6% of poultry and 66.7% of vegetable sellers reporting at least one vaccine dose at the time of the survey. The most common vaccines received were the whole inactivated vaccines (Sinovac or Sinopharm; 44.4%), followed by mRNA vaccines (Moderna or Pfizer; 33.9%). Nobody reported mask use in either group, and only about 1% participants reported a history of a diagnosed COVID-19 (clinical) case in their household before the survey period; 7.9% participants reported a history of asthma, all but one in the poultry workers’ group. Data sparsity meant that these three variables (facemask use, COVID-19 history in household, and asthma) were not explored in further analysis. Details of participants characteristics are in Table 1.

**Table 1:** Characteristics of study participants (N = 291).

Variable	Poultry workers (%)	Vegetable/Fruit sellers (%)	Overall (%)
Sample size	204 (100%)	87 (100%)	291 (100%)
**Sex**			
Male	204 (100%)	87 (100%)	291 (100%)
**Age (median [interquartile range], in years)**	29.5 [22.0; 36.5]	35.0 [24.0; 47.0]	30.0 [23.0; 40.0]
**Age group**			
under 20 years	26 (12.8%)	6 (6.9%)	32 (11.0%)
20-29 years	76 (37.3%)	28 (32.2%)	104 (35.8%)
30-39 years	58 (28.4%)	16 (18.4%)	74 (25.4%)
40-49 years	27 (13.2%)	19 (21.8%)	46 (15.8%)
50+ years	17 (8.3%)	18 (20.7%)	35 (12.0%)
**Religion**			
Muslim	204 (100%)	86 (99.0%)	290 (99.7%)
**Education**			
None	51 (25.0%)	22 (25.3%)	73 (25.1%)
Primary	66 (32.4%)	40 (46.0%)	106 (36.4%)
Secondary or more	87 (42.6%)	25 (28.7%)	112 (38.5%)
**Role in stall**			
Owner	78 (38.2%)	55 (63.2%)	133 (45.7%)
Employee	126 (61.8%)	32 (36.8%)	158 (54.3%)
**Tobacco smoking**			
Non-smoker	86 (42.2%)	32 (36.8%)	118 (40.5%)
Daily smoker	72 (35.3%)	38 (43.7%)	110 (37.8%)
Non-daily smoker	26 (12.7%)	17 (19.5%)	43 (14.8%)
Missing	20 (9.8%)	0 (0.0%)	20 (6.9%)
**Household crowding**			
<2 people per bedroom	46 (22.5%)	16 (18.4%)	62 (21.3%)
≥2-<4 people per bedroom	114 (55.9%)	61 (70.1%)	175 (60.1%)
≥4 people per bedroom	44 (21.6%)	10 (11.5%)	54 (18.6%)
**COVID-19 Vaccine uptake**^a^			
Unvaccinated	64 (31.4%)	29 (33.3%)	93 (32.0%)
Vaccinated (≥1 dose)	140 (68.6%)	58 (66.7%)	198 (68.0%)
**COVID-19 vaccine type**^a^			
Astra-Zeneca	2 (1.4%)	6 (10.3%)	8 (4.0%)
Messenger RNA (Moderna / Pfizer)	52 (37.1%)	15 (25.9%)	67 (33.9%)
inactivated (Sinovac/Sinopharm)	62 (44.3%)	26 (44.8%)	88 (44.4%)
Unknown	24 (17.1%)	11 (19.0%)	35 (17.7%)

a Uptake defined as receipt of at least one vaccine dose (confirmed in vaccination card).

No participants had naso-pharyngeal swabs positive for SARS-CoV-2. Nearly all (99%) participants were positive for anti-S antibodies, whereas the overall prevalence of anti-N antibodies was 59.4% (95% CI 54.7-64.0%), and similar in poultry (59.8%; 95% CI: 55.7-63.7) and vegetable sellers (58.6%; 95% CI: 46.2-70.1) (*P* = 0.890). There was no evidence that the anti-N antibody seroprevalence was associated with any of the participants’ characteristics, except for COVID-19 vaccination. There was some evidence that the anti-N seroprevalence was 55% lower in those who received an mRNA vaccine than unvaccinated participants (crude OR 0.45; 95% CI 0.23-0.85%; *P* = 0.037), with the estimate remaining similar, albeit with a wider CI after adjusting for all other characteristics (adjusted OR 0.49; 95% CI 0.23-1.02; *P* = 0.093). The full results of anti-N seroprevalence and OR of association with participants’ characteristics are in Table 2.

**Table 2:** Seroprevalence of SARS-CoV-2 anti-N IgG antibodies and association to participant characteristics.

Variable	Anti-N antibody prevalence (%) (95% CI)	Crude OR (95% CI)	*P*-value	Adjusted OR^a^ (95% CI)	*P*-value
**Participant group**					
Vegetable sellers (n = 87)	58.6 (46.2-70.1)				
Poultry sellers (n = 204)	59.8 (55.7-63.7)	1.05 (0.63-1.75)	0.851	1.04 (0.58-1.88)	0.89
**Age group**					
under 20 years (n = 32)	65.6 (46.3- 80.9)				
20-29 years (n = 104)	62.5 (54.0-70.3)	0.87 (0.38-2.00)		0.75 (0.28-1.98)	
30-39 years (n = 74)	58.1 (48.1-67.4)	0.73 (0.31-1.72)	0.729	0.70 (0.24-2.02)	0.835
40-49 years (n = 46)	52.2 (34.8-69.0)	0.57 (0.23-1.45)		0.52 (0.16-1.67)	
50+ years (n = 35)	57.1 (39.1-73.5)	0.70 (0.26-1.88)		0.57 (0.17-1.95)	
**Education**					
None (n = 73)	63.0 (47.2-76.4)				
Primary (n = 106)	63.2 (53.5-71.9)	1.00 (0.54-1.87)	0.272	1.08 (0.54-2.17)	0.213
Secondary or more (n = 112)	53.6 (45.0-61.9)	0.67 (0.37-1.24)		0.65 (0.33-1.28)	
**Role in stall**					
Owner (n = 133)	57.1 (49.4-64.5)				
Employee (n=158)	61.4 (57.4-65.2)	1.19 (0.74-1.91)	0.462	1.22 (0.71-1.96)	0.462
**Tobacco smoking**					
Non-smoker (n = 118)	59.3 (49.9-68.1)				
Daily smoker (n = 110)	59.1 (50.6-67.0)	0.99 (0.58-1.68)	0.988	1.12 (0.64-1.96)	0.926
non-daily smoker (n = 43)	60.5 (48.0-71.7)	1.05 (0.51-2.14)		1.09 (0.52-2.30)	
**Household crowding**					
<2 people per bedroom (n = 62)	61.3 (48.8-72.5)				
≥2-<4 people per bedroom (n = 175)	58.8 (52.4-65.0)	0.90 (0.50-1.63)	0.945	0.70 (0.37-1.36)	0.521
≥4 people per bedroom (n = 54)	59.2 (47.5-70.0)	0.92 (0.43-1.94)		0.66 (0.29-1.51)	
**COVID-19 vaccine uptake**^b^					
Unvaccinated (n = 93)	64.5 (48.3-78.0)				
Vaccinated (≥1 dose) (n = 198)	57.1 (51.6-62.3)	0.73 (0.44-1.22)	0.229	0.84 (0.47-1.52)	0.575
**COVID-19 vaccine type**^b^					
Unvaccinated (n = 93)	64.5 (48.2-78.0)	ref		ref	
Astra-Zeneca (n = 8)	50.0 (20.4-79.6)	0.55 (0.13-2.34)		0.65 (0.14-3.05)	
Messenger RNA (BioNTech/Pfizer) (n = 67)	44.8 (33.4-56.7)	0.45 (0.23-0.85)	0.037	0.49 (0.23-1.02)	0.093
Inactivated (Sinovac/Sinopharm) (n = 88)	68.2 (59.7-75.6)	1.18 (0.63-2.18)		1.31 (0.66-2.63)	
Unknown (n = 35)	54.3 (36.9-70.6)	0.65 (0.30-1.44)		0.80 (0.33-1.91)	

a Adjusted for all other characteristics in Table 2.

b Uptake defined as receipt of at least one vaccine dose (confirmed in vaccination card).

## Discussion

In our study, no participant was identified with an active infection with SARS-CoV-2 through polymerase chain reaction testing of the respiratory samples, which correspond with the overall decreasing trend of active SARS-CoV-2 infection reported in Bangladesh during the study period [17]. This also reflected that Bangladesh made good progress in vaccination coverage by the study period and at the beginning of June 2022: more than 68% of Bangladesh's population had received two doses of COVID-19 vaccine [18].

Anti-SARS-CoV-2 S antibodies were near ubiquitous in this study population. With a self-reported vaccine uptake of ∼65%, the findings are likely a combination of naturally acquired and vaccine-derived antibodies, consistent with high levels of transmission during the three COVID-19 pandemic waves before the survey in a population with highly crowded living conditions [19].

Anti-SARS-CoV-2 N antibodies are a short-term proxy for infection that is highly specific for COVID-19 infection, with no cross-reactivity with other related viruses [20,21]. Some seropositive results may be due to inactivated vaccines, although there was no difference in anti-N IgG seroprevalence in participants who had received an inactivated vaccine compared with those who were unvaccinated. Previous studies have also suggested that waning of antibody response with inactivated vaccines is much quicker than mRNA vaccines [22] and anti-N induced by inactivated vaccines have shorter half-life than anti-N from natural infection [23]. Moreover, an earlier study showed a rapid decline in anti-N titers even for post-infection, which is steeper in mild than in severe cases [24]. A lower anti-N seroprevalence was seen in participants with mRNA vaccination, consistent with reported effectiveness against infection [25]. The high seroprevalence (∼60%) was likely due to survey timing at the tail end of the highly infectious Omicron wave [8], a variant that was shown to have good ability to evade naturally acquired immunity from earlier variant infections and vaccine-derived immunity. Studies showed that naturally acquired antibodies for SARS-CoV-2 antigens typically become detectable at a median time of about 2 weeks after the onset of symptoms and start decaying after 4 weeks, depending upon severity [[26], [27], [28]]. A cohort study in the United States reported a half-life for anti-N of 122 days and varied with age [29].

The study finding of high seroprevalence of SARS-CoV-2 among the market workers indicated that probably high levels of transmission had already occurred among the population but that prevention of infection by vaccination was important to top-up natural immunity. Although only ∼1% of participants reported a recent COVID-19 case in the household, it was not surprising considering self-reporting of COVID-19 and testing for symptoms were found low in populations with similar socio-demographic patterns during earlier studies, and high levels of transmission, often asymptomatic or leading to only mild infections, are reflected in the high seroprevalence in cross-sectional studies [6,30]. Seroprevalence assessed between April and October 2020 before vaccination rollouts in slum areas of Dhaka estimated that the seroprevalence using an inhouse anti-S assay was 63.5% [6] and crude seroprevalence of SARS-CoV-2 using a total antibody assay was 60.7% among Forcibly Displaced Myanmar Nationals in Cox's Bazar, Bangladesh during December 2020 [30], both of which have similar living settings to the current study population.

There was some evidence of increased protection from mRNA vaccines compared with other non-whole cell vaccines versions, which was supported by the findings from other earlier studies [31]. We also provide evidence of reasonably good vaccine uptake in manual workers in fixed settings that may indicate the potential for good vaccine coverage in other poor or daily wage populations compared with the national vaccine coverage during the same time [18]. The high seroprevalence reported among wet market workers is not surprising because an earlier study at the beginning of pandemic in China identified a reporting rate for market-to-human transmission that was estimated to be 2-34–fold higher than human-to-human transmission [32]. This high rate informs the need to boost interventions, such as better and greater use of masks. Such a study provides a template and capacity building experience for surveys of the risk and extent of future new and emerging infections in wet markets.

### Limitations

This was a small survey in one set of essential workers in terms of essential food supply and economic drivers of the country. With only 291 participants across 15 markets, limited information on vaccine records and no quantitative measure of antibodies, a formal evaluation of relative vaccine effectiveness by type or antibody kinetics suggestive of declining protection was beyond the scope of this study. We did not have supporting records for the date of vaccination; thus, although we cannot comment on the extent to which some of the anti-N response could have been due to vaccination with whole virus vaccines, any effect would likely be to underestimate any role of vaccination in reducing infection. In addition, a limitation of the Abbott assay includes faster waning of anti-N reactivity than other assays, which would reduce ascertainment of infection and act to underestimate the role of vaccination [33]. In summary, wet market workers appear to be a population at a very high risk of infection by SARS-CoV-2. Given the frontline nature of their job and importance of wet markets, market workers should be considered a priority group for surveillance and control in pandemic preparedness.

## Declarations of competing interest

The authors declare that they have no known competing financial interests or personal relationships that could have appeared to influence the work reported in this paper.
